# Intra- and Inter-rater Reliability of the Lumbar-Locked Thoracic Rotation Test in Patients With Neck Pain

**DOI:** 10.7759/cureus.56407

**Published:** 2024-03-18

**Authors:** Ryota Yoshida, Hironobu Kuruma

**Affiliations:** 1 Department of Physical Therapy, Tokyo Metropolitan University, Tokyo, JPN

**Keywords:** thoracic spine rotation, range of motion, neck pain, intra-rater reliability, inter-rater reliability

## Abstract

Purpose: Neck pain is a common musculoskeletal disorder. Therefore, establishing effective physical therapy for neck pain is one of the most important issues. In addition, in physical therapy for neck pain, it is important to evaluate the thoracic spine, which is an adjacent region of the neck. The lumbar-locked rotation test is designed to evaluate the rotational range of the thoracic spine. However, the reliability of the test when performed on patients with neck pain has not been confirmed.

Objective: We aimed to determine the intra- and inter-rater reliability of the lumbar-locked rotation test in patients with neck pain.

Methods: In this study involving 43 patients, two separate examiners measured thoracic spine rotation. Both examiners conducted three measurements for each side, before and after a five-minute interval. Reliability was assessed using various intra-class correlation coefficient (ICC) models.

Results: The intra-rater reliability showed ICC values of 0.99 for both examiners. The inter-rater reliability showed ICC values of 0.98 for both right and left thoracic rotations.

Conclusion: The findings strongly suggest that the lumbar-locked rotation test has high within-session intra- and inter-rater reliability for patients with neck pain. This test can be considered a reliable method of measuring the thoracic spine rotational range of motion in patients with neck pain in clinical practice.

## Introduction

Neck pain is a common musculoskeletal disorder [1]. In addition to high lifetime and annual prevalence rates of 48.5% and 37.2%, respectively, many people experience recurrent or chronic pain [2]. Furthermore, the socioeconomic impact caused by neck pain on employment is a significant concern, as it often leads to job loss or extended absence due to chronic symptoms. Therefore, establishing effective treatment and prevention methods for neck pain is essential [3,4].

Physical therapy is a standard conservative treatment for neck pain. Effective physical therapy interventions include manual therapy and exercises for the thoracic spine, which have been reported to be beneficial [5]. In addition, a significant relationship between decreased thoracic spinal rotational mobility and neck pain has been observed [6-8], and evaluation of the thoracic spine is recommended for physical therapy in patients with neck pain [9]. Therefore, accurate measurement of the thoracic spine range of motion is crucial for developing a physical therapy treatment plan for neck pain.

A simple and reliable evaluation method is essential to accurately measure thoracic spine range of motion in clinical situations. Johnson et al. investigated the reliability of five methods used to measure thoracic spine range of motion and reported that they all showed high intra-rater reliability [10]. Feijen et al. examined the intra- and inter-rater reliability of the lumbar-locked rotation test, a method used to measure thoracic spine range of motion, and reported good results [11]. However, their study was limited as the results were validated on healthy individuals and could not be directly applied to those with cervical pain [10,11]. Since patients with neck pain may present different characteristics during the measurement procedure compared to healthy individuals, it is crucial to assess the reliability of this measurement technique in patients with neck pain. Therefore, we aimed to determine the intra- and inter-rater reliability of the lumbar-locked rotation test in measuring the range of motion of the thoracic spine in patients with neck pain.

## Materials and methods

This study was performed in accordance with the Guidelines for Reporting Reliability and Agreement Studies (GRRAS) [12].

Participants

Inclusion criteria comprised patients with neck pain, aged between 18 and 60 years, who had a baseline neck disability index [13] score of ≥10/50. Exclusion criteria comprised pregnant or possibly pregnant women, those with a history of spinal surgery, and those who had pain resulting from a motor vehicle accident or from obvious trauma. Patients willing to participate were explained the purpose and content of the study using a research participation explanation document. Those who understood the explanation and agreed to participate in the survey were informed of the measurement procedure and practiced it with an evaluator until it was performed smoothly.

Forty-three (23 females, 20 males) patients participated in the study (mean age, 50.35 ± 8.60 years; mean height, 165.58 ± 7.85 cm; mean weight, 58.79 ± 10.23 kg).

Study design

**Figure 1:**
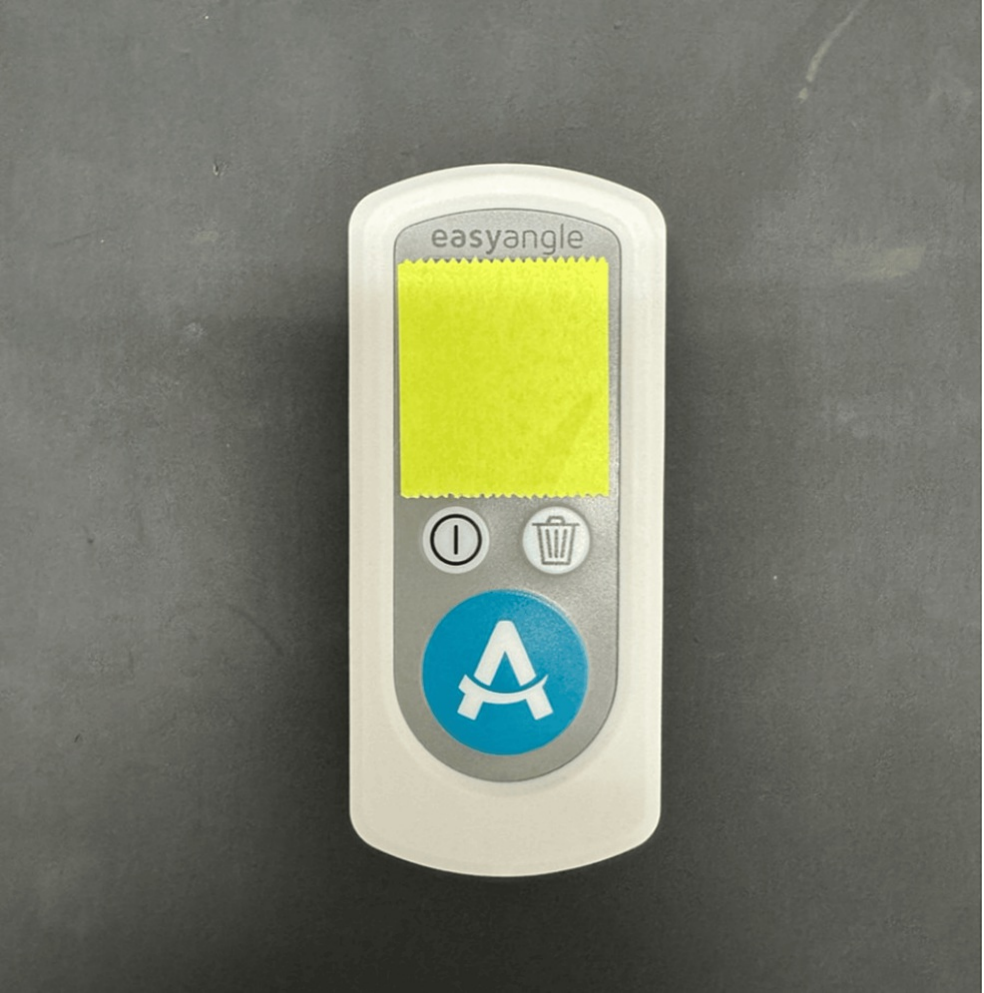
Digital goniometer with a blind monitor* * ITO EasyAngle, Tokyo, Japan

Prior to measurement, Examiner 2 was thoroughly instructed by Examiner 1 on the thoracic spine range of motion measurement method (lumbar-locked rotation test). A digital goniometer (EasyAngle; ITO, Tokyo, Japan) was used to measure the range of rotational motion in the thoracic spine. The measurement accuracy of the digital goniometer was set to 1˚. The digital goniometer monitor displaying the measurements was covered with paper, and the examiners were blinded to the measurement readings (Figure 1). The readings that appeared on the digital goniometer display were read and recorded by a person not involved in the measurements.

Procedure

The patients completed demographic (sex, age, height, and weight) and neck pain questionnaires. The neck pain questionnaire was used to evaluate: (i) pain intensity at rest (numerical pain rating scale, NPRS); (ii) pain intensity at the final range of neck rotation (NPRS); and (iii) a neck disability index (NDI). Subsequently, range of motion in the thoracic spine rotation was measured. Thoracic spinal rotation range of motion measurements were independently performed by Examiners 1 and 2. The order of measurements performed by the examiners was determined randomly, using an envelope method that involved four distinct combinations set prior to the measurements.

Lumbar-Locked Rotation Test

**Figure 2:**
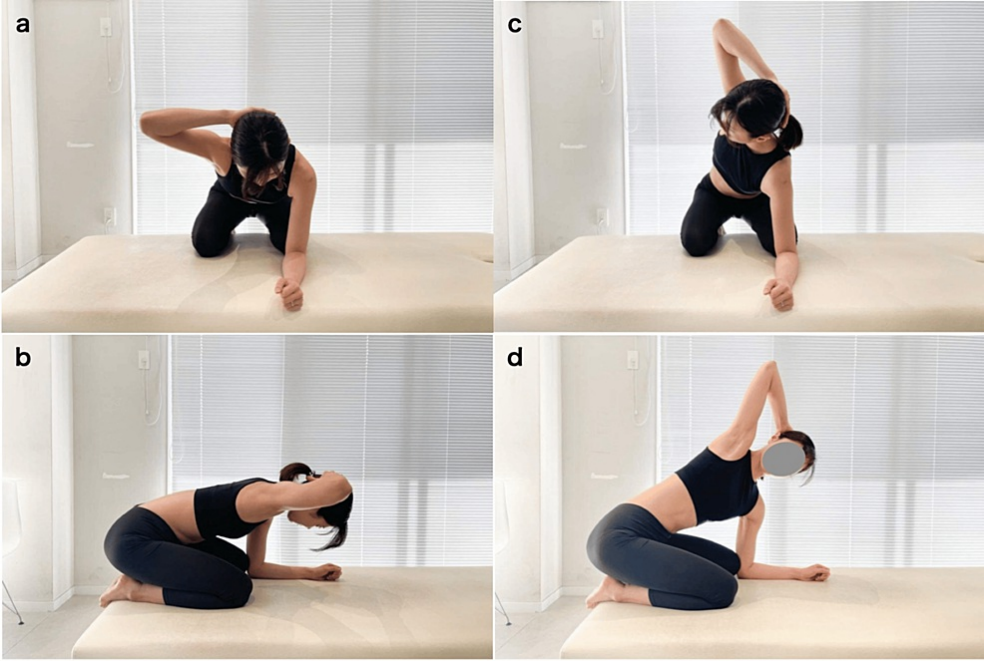
Lumbar-locked rotation test a, b) Starting position for the lumbar-locked rotation test in right thoracic rotation; c, d) End position for the lumbar-locked rotation test in right thoracic rotation

Thoracic rotational mobility was measured using the lumbar spine locking thoracic rotation test protocol (Figure 2) reported by Johnson and Grindstaff [14], with slight modifications. The patients were placed on an examination plinth in maximum knee and hip flexion positions, with the buttocks on the heels, and with their feet out from the edge of the plinth. The patients were placed in a four-point kneeling position, with both elbows in contact with both knees and with the ulnar sides of both forearms and the lateral aspect of the hand in contact with the plinth surface (Figures 2a, 2b). Rotation was performed by placing an ipsilateral hand on the posterior aspect of the neck and rotating the thoracic spine while maintaining a kneeling position (Figures 2c, 2d).

**Figure 3:**
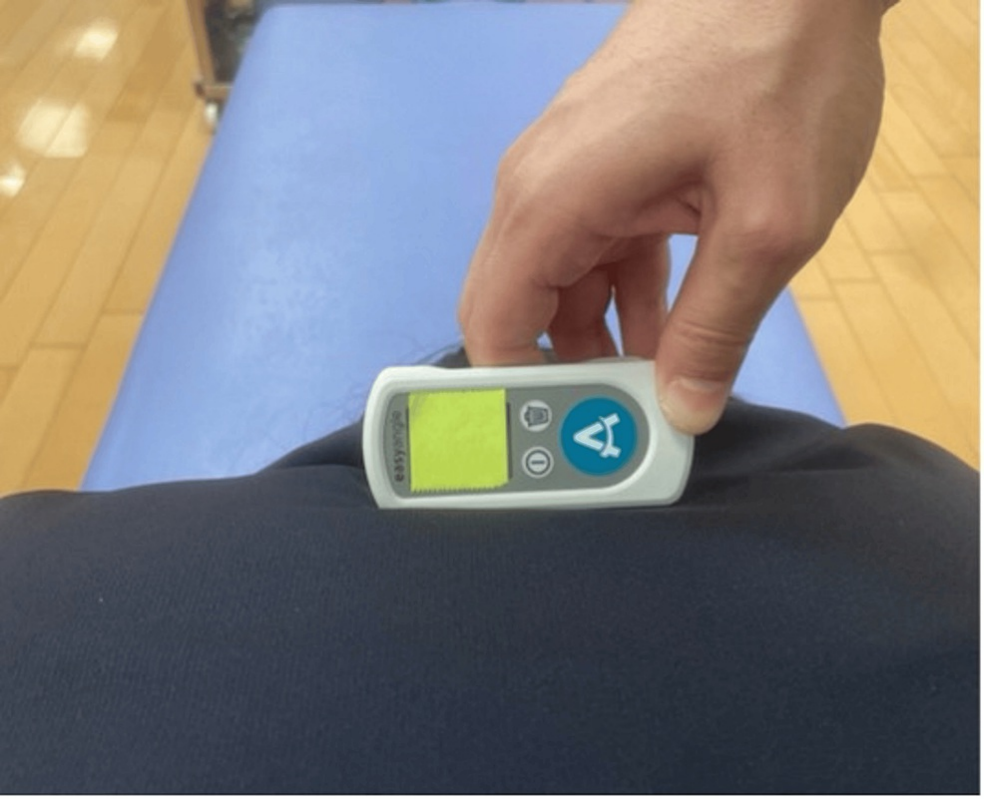
Digital goniometer position

During the measurement process, the digital goniometer was positioned between the scapular spines at the TI-T2 level (Figure 3). The examiner pressed a button on the digital goniometer to record before the initiation of rotation and after the completion of rotation, after which another examiner recorded the readings. Each measurement was performed three times on both the right and left sides.

The criteria for measurement failure were: (i) failure to maintain hip and knee flexion and buttocks away from the heel; (ii) the elbow on the non-rotating side was away from the knee; and (iii) the line connecting the three points of the seventh cervical vertebra, the fifth lumbar vertebra, and the coccyx did not form a straight line. The examiners evaluated the patients based on these three criteria. If a patient was deemed to have failed the procedure based on the abovementioned criteria, the procedure was repeated after a second explanation had been provided.

Statistical Analysis

IBM SPSS Statistics for Windows, Version 27 (Released 2020; IBM Corp., Armonk, New York, United States) was used for statistical analysis. Inter-rater reliability was calculated using intraclass correlation coefficient (ICC) models 2 and 6 (a two-way random-effects model and an absolute agreement model) for the left and right sides, respectively, using the mean of all six trials per side for each assessment. Intra-rater reliability was calculated using ICC models 3 and 3 (two-way mixed-effects models) [14]. The mean of each of the three measurements before and after the five-minute break was compared. Finally, the standard error of measurement (SEM; standard deviation (SD) × √(1-ICC)) and minimum detectable change (MDC) at the 95% confidence level (MDC95, SEM × 1.96 × √2) were used to assess measurement errors related to intra- and inter-rater reliability coefficients. The reliability coefficients were interpreted as follows: <0.50, low; 0.50-0.75, moderate; 0.76-0.90, good; and >0.90, excellent [15].

## Results

The demographic characteristics of the patients are presented in Table 1. There were 23 (67.65%) females and 20 (32.35%) males. The patients ranged in age from 24 to 60 years (mean age, 50.35 ± 8.60 years), had a mean height of 165.58 cm (±7.85), a mean weight of 58.79 kg (±10.23), and an average NDI of 12.63 (±2.02). The mean resting NPRS score was 1.41/10 (±1.06) and the NPRS score in the final cervical rotation range was 5.16/10 (±0.99).

**Table 1:** Participant demographics (mean ± standard deviation) cm, centimeters; kg, kilogram; n, number (of patients); NDI, neck disability index; NPRS, numerical pain rating scale

Characteristics	Females (n = 23)	Males (n = 20)	Total (n = 43)
Age (years)	47.48（±9.89）	56.65（±5.57）	50.35 (±8.60)
Height (cm)	160.22（±5.66）	171.75（±5.13）	165.58 (±7.85)
Weight (kg)	51.57（±6.35）	67.10（±7.18）	58.79 (±10.23)
NDI	12.13（±2.05）	13.20（±1.89）	12.63 (±2.02）
NPRS at rest	1.22（±1.04）	1.60（±1.07）	1.40 (±1.06）
NPRS during maximum neck rotation	4.91（±0.95）	5.45（±0.97）	5.16 (±0.99)

The mean values (±SD) of the lumbar-locked rotation tests used for within-session intra- and inter-rater reliability analyses are presented in Tables 2, 3, respectively. Table 4 shows the intra- and inter-rater ICC with 95% confidence intervals (CI).

**Table 2:** Mean values (±standard deviation) of both Examiners’ first and second lumbar-locked rotation tests

		Examiner 1			Examiner 2	
		Procedure 1	Procedure 2		Procedure 1	Procedure 2
Lumbar-locked rotation test (°)	Right	41.45 ± 10.04	41.74 ± 10.09		42.26 ± 10.26	42.31 ± 10.15
	Left	40.62 ± 8.03	40.73 ± 8.12		41.49 ± 8.23	41.48 ± 8.06

**Table 3:** Mean values (±standard deviation) of both Examiners’ six trials per side of rotation

		Right		Left
Lumbar-locked rotation test (°)	Examiner 1	41.60 ± 10.05		40.67 ± 8.05
	Examiner 2	42.29 ± 10.19		41.48 ± 8.12

**Table 4:** Within-session intra-rater reliability (ICC 3,3) and inter-rater reliability (ICC 2,6) CI, confidence interval; ICC, intra-class correlation coefficient (average measure); SEM¼, standard error of measurement; MDC95, minimal detectable change with a 95% CI

		Right			Left		
		ICC (95% CI)	SEM (°)	MDC95(°)	ICC (95% CI)	SEM (°)	MDC95(°)
Intra-rater	Examiner 1	0.99 (0.995–0.999）	1.01	2.80	0.99 (0.993–0.998)	0.81	2.24
	Examiner 2	0.99 (0.992–0.998)	1.02	2.83	0.99 (0.994–0.998)	0.81	2.26
Inter -rater	Examiners 1-2	0.98 (0.970–0.992)	1.43	4.00	0.98 (0.960–0.992)	1.14	3.17

Intra-rater reliability analysis indicated that the ICC values for right thoracic and left thoracic rotations for Examiner 1 were 0.99 (95% CI 0.995-0.999) and 0.99 (95% CI 0.993-0.998), respectively. The ICC values for right thoracic and left thoracic rotation for Examiner 2 were 0.99 (95% CI 0.992-0.998) and 0.99 (95% CI 0.994-0.998), respectively. Using this reliability analysis, we compared the means of three measurements before and after a five-minute break for each left and right side.

Within-session inter-rater reliability analysis of the lumbar-locked rotation test showed ICC values between 0.98 (95% CI 0.970-0.992) for right thoracic rotation and 0.98 (95% CI 0.96-0.992) for left thoracic rotation. This reliability analysis used the examiners’ mean values for all six trials on each rotation side.

## Discussion

The ICC of the measurements obtained in this study showed that the lumbar-locked rotation test was reliable for measuring the rotational range of motion in the thoracic spine in patients with neck pain. The thoracic spine rotation angles obtained in this study were similar to those reported by Johnson et al. in healthy adults. While the patients in this study had cervical pain, the lumbar-locked rotation test did not cause pain, and pain did not affect the results or the reliability of the test.

The intra-rater ICC was 0.99 for Examiners 1 and 2, indicating excellent reliability. The SEM ranged from 0.81 to 1.02 for intra-rater reliability and from 1.14 to 1.43 for inter-rater reliability. The MDC95 ranged from 2.24 to 2.83 for intra-rater reliability and from 3.17 to 4.00 for inter-rater reliability.

Johnson et al. examined the intra-rater reliability of the lumbar-locked rotation test in 46 healthy adults and reported an ICC ranging from 0.86 to 0.90, an SEM ranging from 2.0 to 2.1, and an MDC ranging from 5.53 to 5.89 [10]. In a similar study of young swimmers by Feijen et al., the ICC for intra-rater reliability ranged from 0.91 to 0.96, the SEM ranged from 3.1 to 3.8, and the MDC ranged from 8.59 to 10.56; the ICC for inter-rater reliability ranged from 0.86 to 0.89, the SEM ranged from 4.41 to 5.02, and the MDC ranged from 12.22 to 13.91 [11]. The results obtained in this study showed higher reliability than those previously reported. This difference in the reported measurements can be attributed to the devices utilized for the measurements and the procedure to perform the lumbar-locked rotation test. In our study, we measured the thoracic spine rotation range of motion using a digital goniometer that digitally displayed measurements in 1˚ increments. Hwang et al. examined the reliability of the lumbar-locked rotation test using goniometers, bubble inclinometers, dual digital inclinometers, and smartphone inclinometers [16]. They reported that the digital inclinometer and smartphone clinometer measurements were more reliable than those obtained using a goniometer and a bubble inclinometer. In addition, in previous studies, failure criteria for the lumbar-locked rotation test included the inability to assume the quadruped position, loss of lumbar spine position, scapular retraction, and loss of upper extremity position [10,11,14,16]. However, these criteria are highly subjective and contain ambiguous elements.

In contrast, in our study, the failure criteria were: (i) the buttocks away from the heels; (ii) the elbow on the non-rotating side away from the knee; and (iii) the line connecting the three points of the seventh cervical vertebra, fifth lumbar vertebra, and coccyx not forming a straight line. During the pre-measurement practice phase, we confirmed that there were no differences between Examiners 1 and 2 in determining whether the line connecting the three points was straight. These criteria were considered to likely avoid compensatory movements such as lateral flexion and extension of the trunk and increase the reproducibility of movements. In addition, the previous study did not describe patients’ pre-measurement warm-up or practice. However, in our study, the patients practiced sufficiently to ensure that they could perform the movements consistently, which we consider was one reason for the reduced inter-rater variability. Our study findings suggest that the lumbar-locked rotation test can be reliably used in clinical practice, even for patients with neck pain.

## Conclusions

In this study, the reliability of the lumber-locked rotation test was tested on patients with neck pain. Inter-rater reliability was calculated using the mean of all six trials, left and right, respectively, using the ICC models 2 and 6. Intra-rater reliability was calculated using ICC models 3 and 3. The results of this study suggest that within-session intra- and inter-rater reliability of the lumbar-locked rotation test for patients with neck pain is good to excellent (0.98 for inter-rater ICC, 0.98; intra-rater ICC, 0.99), indicating that this test can be used reliably in clinical practice.
